# Blunt Abdominal Trauma among Patients Admitted to the Department of Surgery at a Tertiary Care Centre: A Descriptive Cross-sectional Study

**DOI:** 10.31729/jnma.8154

**Published:** 2023-05-31

**Authors:** Roshan Ghimire, Bidur Prasad Acharya, Prashanta Pudasaini, Yugal Limbu, Dhiresh Kumar Maharjan, Prabin Bikram Thapa

**Affiliations:** 1 Department of Surgery, Kathmandu Medical College and Teaching Hospital, Sinamagal, Kathmandu, Nepal

**Keywords:** *blunt injuries*, *conservative management*, *operative surgical procedure*

## Abstract

**Introduction:**

Blunt abdominal trauma bears significant morbidity and mortality worldwide and needs careful evaluation and management for a better outcome, where the resources are limited and the impact of the financial burden is very important. Previously, many cases used to be managed with operative procedures, and now the trend has been shifting to non-operative management. This study aimed to determine the prevalence of blunt abdominal trauma among patients admitted to the Department of Surgery of a tertiary care centre.

**Methods:**

This was descriptive cross-sectional study done between 1 February 2022 to 31 January 2023 after taking ethical approval from the Institutional Review Committee (Reference number: 2312202103). The decision of non-operative versus operative treatment was decided with dynamic clinical evaluation and severity of intraabdominal injuries. Demographic data, the mechanism of injury, and both conservative and operative management were studied. All the patients who were more than 18 years of age, and admitted to the Department of Surgery were included in the study. Convenience sampling method was used. Point estimate and 95% Confidence Interval were calculated.

**Results:**

Among a total of 1450 patients, the prevalence of blunt abdominal trauma was 140 (9.65%) (8.13-11.17, 95% Confidence Interval). A total of 61 (43.57%) were young within the age group of 18-30 with a male-female ratio of 4:1. Road traffic accidents 79 (56.43%) were the most common mechanism followed by falls from heights 51 (36.43%).

**Conclusions:**

The prevalence of blunt abdominal trauma among patients admitted to the Department of Surgery was found to be higher than in other studies done in similar settings.

## INTRODUCTION

In developing countries, trauma is one of the causes of morbidity and mortality and the leading cause of death, especially in people under the age of 45 years.^1^ The variability in clinical findings makes it difficult and challenging to diagnose blunt abdominal trauma even by experienced surgeons.^2^

It creates various ranges of mild, single-system to multisystem injuries so to minimize morbidity and mortality, decisions should be made early for their detection.^3^ In the last two decades the notable change in the management of blunt abdominal trauma is the shift from routine operative to non-operative management (NOM) and NOM of blunt solid organ injury has become the rule rather than the exception.^4^ The important cause of morbidity and mortality in Nepal is trauma-related injuries, which is the third leading cause.^5^

The aim of this study was to find out the prevalence of blunt abdominal trauma among patients admitted to the Department of Surgery of a tertiary care centre.

## METHODS

A descriptive cross-sectional study was done among patients admitted to the Department of Surgery of Kathmandu Medical College Teaching Hospital, Sinamangal, Kathmandu, Nepal from 1 February 2022 to 31 January 2023. The ethical approval was taken from the Institutional Review Committee of Kathmandu medical college and teaching hospital (Reference number: 2312202103). Informed consent was obtained from all patients for the utilization of their data for research purposes. All the patients who were more than 18 years of age, and admitted to the Department of Surgery were included in the study. Patients leaving against medical advice (LAMA), having pregnancy, and having an unambiguous focused assessment with sonography for trauma (FAST) scans were excluded from the study. Convenience sampling was done. Advanced Trauma Life Support (ATLS) protocol was used to assess each patient. The sample size was calculated by using the following formula:


$$\begin{array}{ll} \text{n} & ={\text{Z}}^{2}\times \frac{\text{p}\times \text{q}}{{\text{e}}^{\text{2}}}\\ & =1.96^{2}\times \frac{0.50\times 0.50}{0.03^{2}}\\ & =1067 \end{array}$$

Where,

n = minimum required sample size
Z= 1.96 at 95% Confidence Interval (CI)
p = prevalence taken as 50% for maximum sample size calculation
q = 1-p
e = margin of error, 3%

The calculated sample size was 1067. After adding 10% non-response rate, the sample size was 1186. However, 1450 sample size was taken.

Patients after the presentation were resuscitated first followed by detailed clinical history, physical examinations, laboratory (complete blood count, renal function, liver function, coagulation profile, amylase, blood sugar, sodium, and potassium level), and radiological tests FAST and contrast-enhanced computed tomography (CECT) to come to the diagnosis and were categorized as stable or unstable. Detail of gender, age, time of presentation to hospital, mechanism of injury, FAST scan, CECT report, associated injuries, and intraoperative findings was taken. Patients levelled as true positives and negatives based on the collective findings on imaging. Those with FAST positive and CECT negative were levelled as "false positive" and those with negative FAST and positive CECT were levelled as "false negative".

Patients were also categorized using the blunt abdominal trauma scoring system (BATSS) which is a 24-point scoring system including abdominal pain (2 points), abdominal tenderness (3 points), chest wall sign (1 point), pelvic fractures (5 points), positive FAST (8 points), systolic blood pressure <100 mmHg (4 points) and pulse rate >100 (1 point) with division into low risk (<8 points), moderate (8-11 points) and high risk ≥12 points.^1^

Dynamic physical examination was carried out to know the progress of patients with close monitoring. Then the decision was taken whether to operate or not depending on the response to conservative management, clinical deterioration despite adequate resuscitation, and severity of intraabdominal pathology like hollow viscus perforation, splenic laceration, etc which were straight forward requiring urgent operative management.

Data were entered in Microsoft Excel 2016 and analysis was done using IBM Statistics SPSS 20.0. Point estimate and 95% CI were calculated.

## RESULTS

Among a total of 1450 patients, the prevalence of blunt abdominal trauma was 140 (9.65%) (8.13-11.17, 95% CI). The male-to-female ratio is 4:1. Total of 61 (43.57%) was under the age group of 18-30 years. Rib fractures 24 (17.14%) and hemothorax 24 (17.14%) were the most commonly associated with extra-abdominal injuries. Pelvic and spine fractures were seen in 7 (5%) and 6 (4.29%) cases. Splenic injury is the commonest solid organ to be involved 56 (40%). Among hollow viscus, the small intestine 17 (12.14) was commonly injured. In about 8.57% of cases, multiple organs 12 (8.57%) were involved (Table 1).

**Table 1:** Patient demographics (n = 140).

Variables	(n%)
**Age (years)**	
18-30	61 (43.57)
31-50	28 (20)
51-70	34 (24.29)
>70	17 (12.14)
**Sex**	
Male	112 (80)
Female	28 (20)
**Distribution of cases**	
Spleen	56 (40)
Liver	50 (35.71)
Kidney	22 (15.71)
Small intestine	17 (12.14)
Abdominal wall	16 (11.43)
Mesenteric tear	7 (5)
Retroperitoneal hematoma	6 (4.29)
Pancreas	4 (2.85)
Urinary bladder	3 (2.14)
Stomach	2 (1.43)
Vascular	2 (1.43)
**CT findings**	
Solitary solid organ	106 (75.71)
Abdominal wall	16 (11.43)
Hollow viscus	14 (10)
Multiple solid organs	12 (8.57)
Mesentery	7 (5)
Vascular injury	1 (0.71)
**Associated injuries**	
Hemothorax	24 (17.14)
Rib fractures	24 (17.14)
Head injury	19 (13.57)
Pneumothorax	18 (12.86)
Extremities fracture	17 (12.14)
Pelvis fracture	7 (5)
Spine fracture	6 (4.29)

**Figure 1:**
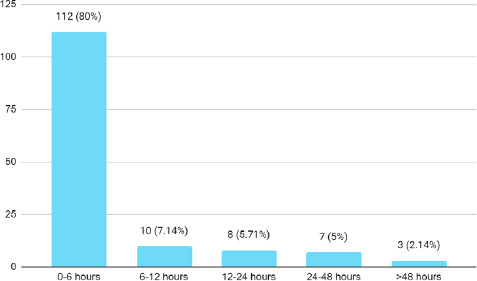
Time of presentation by a number (n= 140).

The mechanism of injuries were road traffic accidents 79 (56.43%) followed by fall from height 51 (36.43%) and 5 (3.58%) each of physical assault and industrial injuries. The majority of the patient presented with pain abdomen followed by vomiting and chest pain. Hypovolemic shock was present in 22 (15.71%) patients. Most of the patients 112 (80%) presented within 6 hours of the incident (Figure 1).

A total of 114 (81.42%) were managed conservatively and 26 (18.57%) underwent surgery. A total of 4 (2.86%) were unstable. The commonest surgery performed was resection and anastomosis 8 (30.78%) followed by splenectomy 3 (11.54%) (Table 2).

**Table 2:** Procedure performed (n= 26).

Procedures	n (%)
Resection n anastomosis	8 (30.78)
Splenectomy	3 (11.54)
Primary bowel repair	3 (11.54)
Duodenorrhaphy with pyloric exclusion with retro colic GJ with the biliary diversion with T-tube placement	2 (7.68)
Abdominal wall closure	2 (7.68)
Bladder repair	2 (7.68)
Spleen preserving distal pancreatectomy with primary repair of left renal and splenic vein	1 (3.85)
Total pancreatectomy	1 (3.85)
Control of bleeding from presacral plexus	1 (3.85)
Repair of perianal tear	1 (3.85)
Mesenteric repair	1 (3.85)
Primary repair of gastric perforation	1 (3.85)

Out of total 26 (18.57%) patient undergoing major surgery, only 1 (5.88%) was on a low-risk. and 3 (7.69%) cases operated for moderate risk and 22 (26.19%) patients operated with high-risk scores (Table 3).

**Table 3:** Blunt Abdominal Trauma Severity Score (BATSS) with the procedure (n= 140).

BATSS	Low risk (<8)	Moderate risk (8-11)	High risk ≥12
	17 (12.14)	39 (27.86)	84 (60)

The mean length of intensive care and hospital stay was 2.6 days and 7.8 days respectively. The total mortality was 3 (2.14%). The mortalities had severe degrees of extra-abdominal injuries making them difficult to manage. Among them, 1 (0.71%) had bilateral severe lung contusion with cardiac contusion with grade-I splenic injury with hemoperitoneum with gastric perforation, 1 (0.71%) had bilateral hemopneumothorax with multiple rib fractures with severe lung contusion with AAST grade-IV liver injury, and 1 (0.71%) had Bilateral multiple rib fractures with hemopneumothorax with moderate lung contusions with American Association for Surgery of Trauma (AAST) grade-V pancreatic injury (Table 4).

**Table 4:** Morbidities (n = 22).

Morbidities	n (%)
Septicemia	4 (2.86)
Intestinal obstruction	3 (1.53)
SSI^*^	3 (1.53)
Wound dehiscence	3 (1.53)
Pneumonitis	2 (1.43)
DVT^†^	2 (1.53)
UTI^‡^	1 (0.71)
ARDS^§^	1 (0.71)
Bed sores	1 (0.71)
Enterocutaneous fistula	1 (0.71)
Hospital-acquired pneumonia	1 (0.71)

* SSI= Surgical site infection

† DVT = Deep vein thrombosis

‡ UTI = Urinary tract infection

§ ARDS= Acute respiratory distress syndrome

## DISCUSSION

In our study, the prevalence of blunt abdominal trauma was 9.66% which is comparable with a study.^6^ Among blunt abdominal trauma patients, the age group between 18-30 years were common (43.57%) with male predominance (80%) presenting within 6 hours of the incident (80%).^6^ The abdomen is the third most commonly injured region affecting about 7-10% of trauma patients with almost 85% being blunt in nature which can be isolated or associated with other injuries.^7^ Blunt abdominal trauma is one of the leading causes of morbidity and mortality in young adults commonly caused by road traffic accidents followed by falls from heights and assaults.^7^ Depending on the velocity of injury the severity might be diverse, ranging from simple abdominal abrasions to life-threatening organ injury which needs prompt intervention and even with best efforts might even result in death.^8^

During the presentation, it is vital to understand the pathophysiology, establish the diagnosis early, and start intervention right from the beginning to have a better outcome. Non-operative management has been successful in many cases but operative management plays a vital role in cases where emergent surgery can save lives and early referral to the high-volume centre if presented to the low-resourceful centre is important.^9^ Among the prevalence of abdominal injuries spleen (40%) was the commonest followed by the liver (35.71%) and small intestine (12.14%) which was consistent with findings noted by a study.^10^ Single solid organ injuries were prevalent rather than multiple organs. Among hollow viscus, ileal perforation was mostly seen. Hemothorax and rib fractures are mostly associated with blunt abdominal trauma and head injuries and fractures of the extremities.^6^

To perform the surgery with safety and less morbidity is always crucial but depending on the gravity of the injury it is not always ideal to achieve what is expected. There was a total of 3 (2.14%) mortalities including two operated cases and one conservatively managed case among the mortalities, multiple organ involvement during the initial insult was seen which is similar to the findings by one of the studies.^11^ Refractory septic and hemorrhagic shock were key components along with pulmonary and cardiac problems like contusions, hospital-acquired pneumonia, and acute respiratory distress syndrome. The time of presentation was key in most of the cases as those presented earlier had better overall outcomes in comparison to those who presented late. Wound management plays an integral role in postoperative hospital stay and overall cost. There were a few cases of pneumonitis and one each of deep vein thrombosis, bed sore, hospital-acquired pneumonia, entero-cutaneous fistula, acute respiratory distress syndrome, and urinary tract infection during the management of these trauma cases.

## CONCLUSIONS

The prevalence of blunt abdominal trauma among patients admitted to the Department of Surgery was found to be similar in other studies done in similar settings. Initial resuscitation, clinical examination, and establishment of correct diagnosis and management plan maybe the key to the management. The time of presentation is vital to their outcome with early diagnosis and treatment can save many lives. Though most of the cases can be managed conservatively, the decision and correct timing of operative surgery hold much importance.
